# Supplementary material for: The burden of stillbirths in low resource settings in Latin America: Evidence from a network using an electronic surveillance system
Source: PLoS One. 2023 Dec 22;18(12):e0296002. doi: 10.1371/journal.pone.0296002 (PMC10745214; doi:10.1371/journal.pone.0296002)
Supplement: S1 File — Independent variables considered in the analysis. S2 Table. The WHO criteria for potentially life-threatening conditions and maternal near miss. (DOCX) [file pone.0296002.s002.docx]

**Table S1**. Independent variables considered in the analysis

| **Variables** | **Definition/categories** |
| --- | --- |
| **1. Maternal characteristics** |  |
| Age | defined as age of the mother in complete years at the time of delivery, categorized into 3 groups: less than 19, 20 to 34 and more than 34 years |
| Ethnicity | skin color, categorized as white, indigenous, black or pardo, and others |
| Literacy | categorized as no, if illiterate and yes if there was any degree of education, whether secondary or university |
| Marital status | dichotomized between married women or those in a stable union or single women with no partner |
| Parity | number of previous births, including stillbirths |
| Previous stillbirths | having had a previous stillborn |
| Anaemia | y/n |
| Diabetes | y/n |
| Hypertension | y/n |
| Preeclampsia | y/n |
| Eclampsia/HELLP | y/n |
| Cardiac disease | y/n |
| Renal disease | y/n |
| **2. Pregnancy characteristics** |  |
| ANC screening for Toxoplasmosis | categorized in done, partially done, not done |
| ANC screening for Syphilis | categorized in done, partially done, not done |
| ANC screening for HIV | categorized in done, partially done, not done |
| **3. Pregnancy and neonatal outcomes** |  |
| Mode of delivery | c-section, spontaneous or operative vaginal delivery |
| Placental abruption | y/n |
| Congenital anomalies | y/n |
| Sex/gender | Gender of the newborn, categorized in male or female |
| Health professionals assisting childbirth | health professional who assisted woman during childbirth, categorised as a medical doctor or obstetrician, nurse or other. |
| Gestational age at birth | gestational age at delivery |
| Birth weight | newborn birth weight |
| **4. Maternal morbidity** |  |
| Potentially life-threatening conditions (PLTC) | clinical conditions that threaten a woman’s life during pregnancy, labor and the postpartum period |
| Maternal near-miss (MNM) | woman who nearly died but survived a complication that occurred during pregnancy, childbirth or postpartum |
| Maternal death (MD) | woman who died during pregnancy or postpartum periods |
| Severe maternal outcome (MNM+MD) | sum of MNM and maternal death cases |

**Table S2.** The WHO criteria for potentially life-threatening conditions and maternal near miss

1. **Criteria for potentially life-threatening conditions**

**Hemorrhagic disorders**

Abruptio placentae

Accreta/increta/percreta placenta

Ectopic pregnancy

Postpartum Hemorrhage

Ruptured uterus

**Hypertensive disorders**

Severe preeclampsia

Eclampsia

HELLP syndrome

**Other systemic disorders**

Endometritis

Seizures

**Cont. Other Systemic disorders**

Sepsis

Shock

Thrombocytopenia <50.000

Thyroid crisis

**Severe Management Indicators**

Blood transfusion

Central venous access

Hysterectomy

ICU admission

Prolonged hospital stay (>7 postpartum days)

Non-anesthetic Intubation

Surgical intervention

1. **Criteria for maternal near miss criteria**

**Clinical criteria**

Acute cyanosis

Gasping

Respiratory rate >40 or <6/min

Shock

Oliguria non responsive to fluids or diuretics

Clotting failure

Loss of consciousness lasting ≥12 hours

Loss of consciousness AND absence of pulse/heart beat

Stroke

Uncontrollable fit/total paralysis

Jaundice in the presence of pre-eclampsia

**Laboratory-based criteria**

Oxygen saturation <90% for ≥60 minutes

pH <7.1

**Cont. Laboratory-based criteria**

PaO2/FiO2 <200 mmHg

Lactate >5

Creatinine ≥300 mmol/l or ≥3.5 mg/dl

Acute thrombocytopenia (<50 000 platelets)

Bilirubin >100 mmol/l or >6.0 mg/dl

Loss of consciousness AND the presence of glucose and ketoacidosis in urine

**Management-based criteria**

Use of continuous vasoactive drugs

Hysterectomy following infection/hemorrhage

Transfusion of ≥5 units red cell transfusion

Intubation and ventilation for ≥60 minutes not related to anesthesia

Dialysis for acute renal failure

Cardio-pulmonary resuscitation (CPR)

1. **Severe Maternal Outcome**

Maternal near miss or maternal death.

1. **Maternal Death**

Maternal death during hospitalization or before return to the postnatal visit.
