# Supplementary material for: The burden of stillbirths in low resource settings in Latin America: Evidence from a network using an electronic surveillance system
Source: PLoS One. 2023 Dec 22;18(12):e0296002. doi: 10.1371/journal.pone.0296002 (PMC10745214; doi:10.1371/journal.pone.0296002)
Supplement: Attachment — Submitted filename: Comments on PLOS One.docx [file pone.0296002.s003.docx]

Thank you for this article which provides us very valuable information on stillbirth rates, risk factors and regional differences of five Latin American countries. The authors analyzed a large dataset of almost 100000 births.

General comments

- Article should be proofreaded by a native English speaker.
- Text must be more concise and objectives stated more clearly
- Focus on the flow of the article and apply more structure in the text
- With the intention to compare data I would advise to use ICD-PM and the definition of the WHO

Comments

1. Abstract

- Are we reporting stillbirth rates (line 33) or ratio’s line 46) ? The WHO uses as denominator live births and stillbirths ( ref <https://www.who.int/docs/default-source/mca-documents/maternal-nb/making-every-baby-count.pdf>.). Please also read <https://www.ncbi.nlm.nih.gov/pmc/articles/PMC6631739/> on how to define stillbirths and what to use as denominator, don’t use rate and ratio’s as they are the same.
- In the objective in the abstract line 33 -34 the authors state that they determine the stillbirth rate and the regional, maternal end pregnancy characteristics and assiociated factors. Do they mean risk factors associated with stillbirths?
- Be more specific in what exactly your objectives are ( -determine stillbirth rate, analyze the association with maternal, perinatal and delivery characteristics and geographical differences?)
- In the abstract we don’t see any conclusion about the regions. If you state it in the objective you have to say something about it in the result/conclusion
- In line 36 please add SIP as abbreviation for the first time

2. Introduction

- line 66-67 please correctly use what definition the reference uses – 22 weeks and not 20 weeks is the threshold – early and late stillbirth definition.
- Start the introduction with relevant information about the worldwide burden is of stillbirth (first paragraph) and than continue to Latin America, now the first 4 lines is only about the definition and this drags the attention away from the article
- Line 72 uses now FDR – it is better to not use several terms like rate and ratios stillbirth and fetal death without giving explanation…. Please read the article <https://www.ncbi.nlm.nih.gov/pmc/articles/PMC6631739/> on how to define stillbirths and what to use as denominator, don’t use rate and ratio’s as they are the same.
- Line 78 “3 million maternal, fetal and neonatal deaths….” Is unclear what is 3 million….
- Be aware to be consistent in the definition of stillbirth rate (WHO – denominator is the total births)
- Line 97-101 is too long for one sentence and by referring to the article you can summarize what you want to say – stillbirth rates and the annual reduction are very heterogenous on … and than summarize .. because all the figures in the introduction makes it too long.
- Relate these differences in stillbirth rate in the Latin American Caribbean region with the objective of your study so that we can better understand the relevance of this study.
- Line 104 – is it contributing or **could** contribute? Are the countries doing this now? Do you have a reference than where individual countries do that?

3. Material and methods

- Please use the strobe checklist and add more structure to the methods section and provide all information accordingly such as describe the type of study etc. <https://www.strobe-statement.org/checklists/>
- Line 121-142 can be made better understandable with a flowchart or table focusing on 1. Maternal characteristics ( age, ethnicity….) 2. pregnancy characteristics (…..), 3. delivery characteristics (…..)
- Line 146 – stillbirth ratio – and total live birth is used as denominator…. This should be more specific and comparible – so please use WHO criterio so that the data can be compared!
- I would suggest, although you don’t have the causes of death to use the ICD-PM to be able to compare data ( <https://pubmed.ncbi.nlm.nih.gov/35972943/> , <https://pubmed.ncbi.nlm.nih.gov/32777997/> ).
- Maternal morbidity (MNM, PTLC, SMO, MD) : who attributed maternal morbidity to a case? Who defined a case as a MNM, PTLC etc? and how accurate is the database on this, who classified the case as …..What is the reason the authors choose for this (MNM, PTLC, SMO, MD)
- Is it possible to group the maternal condition according to ICD-PM groups M1-M5

4. Results

- Line 172, do not mention definition again

5. Discussion

- Mention your main results, after line 209 also report the other findings shortly
- Discuss than those findings the authors started with geographical differences, try to explain why some countries might have higher stillbirth rates….
- Than discuss the relevant maternal (hypertension, diabetes, infection) pregnancy and delivery outcomes (as is
- Line 255-260 – we do not have any results on BMI, in the discussion discuss your own findings….
- One limitation is that there are no causes attributed to the stillbirth cases
- Line 313 -314 the conclusion that the SIP is a tool of utmost importance is not in the scope of this study
