# Supplementary material for: The burden of stillbirths in low resource settings in Latin America: Evidence from a network using an electronic surveillance system
Source: PLoS One. 2023 Dec 22;18(12):e0296002. doi: 10.1371/journal.pone.0296002 (PMC10745214; doi:10.1371/journal.pone.0296002)
Supplement: Attachment — Submitted filename: Response to Reviewers.docx [file pone.0296002.s004.docx]

Campinas, July 15th 2023

_____________________________________________________________________

Thank you for this article which provides us with very valuable information on stillbirth rates, risk factors and regional differences in five Latin American countries. The authors analyzed a large dataset of almost 100000 births.

General comments

- Article should be proofreaded by a native English speaker.
- Text must be more concise and objectives stated more clearly
- Focus on the flow of the article and apply more structure in the text
- With the intention to compare data I would advise to use ICD-PM and the definition of the WHO

Comments

1. Abstract

- Are we reporting stillbirth rates (line 33) or ratio’s line 46) ? The WHO uses as denominator live births and stillbirths ( ref <https://www.who.int/docs/default-source/mca-documents/maternal-nb/making-every-baby-count.pdf>.). Please also read <https://www.ncbi.nlm.nih.gov/pmc/articles/PMC6631739/> on how to define stillbirths and what to use as denominator, don’t use rate and ratio’s as they are the same.

Response: Thank you for raising this point and the opportunity to make it clearer. We intended to estimate stillbirth ratios (the number of fetal deaths per 1,000 live births), although some of our referenced papers had analysed stillbirth rates (number of fetal deaths per 1,000 total births, including live births and fetal deaths). We included a clarification about the difference between stillbirth ratio and rate in the introduction and a rationale about the different approaches. In our analysis, we made clear that we estimated stillbirths using stillbirth ratio, as defined by the National Center for Health Statistics of the USA.

- In the objective in the abstract line 33 -34 the authors state that they determine the stillbirth rate and the regional, maternal end pregnancy characteristics and associated factors. Do they mean risk factors associated with stillbirths?

Response: Yes, we meant to say “risk-factors”. The objectives were changed to “To determine recent stillbirth ratios, and analyse the association with maternal, perinatal and delivery characteristics and geographical differences and its risk factors in some Latin American countries (LAC)”.

- Be more specific in what exactly your objectives are ( -determine stillbirth rate, analyze the association with maternal, perinatal and delivery characteristics and geographical differences?)

Response: Yes, thank you for giving this suggestion; it was exactly what we meant to say. Therefore, we changed the objectives accordingly (see below).

- In the abstract we don’t see any conclusion about the regions. If you state it in the objective you have to say something about it in the result/conclusion

Response: We added the following conclusion to address the regional differences: The stillbirth ratios varied across the maternity hospitals, which highlight the importance for individual surveillance.

- In line 36 please add SIP as abbreviation for the first time

Response: Thank you. The abbreviation was added accordingly.

2. Introduction

- line 66-67 please correctly use what definition the reference uses – 22 weeks and not 20 weeks is the threshold – early and late stillbirth definition.

Response: Thank you. Changes were made accordingly.

- Start the introduction with relevant information about the worldwide burden is of stillbirth (first paragraph) and then continue to Latin America, now the first 4 lines is only about the definition and this drags the attention away from the article

Response: We changed the sequence of paragraphs in the introduction to improve the flow and its readability.

- Line 72 uses now FDR – it is better to not use several terms like rate and ratios stillbirth and fetal death without giving explanation…. Please read the article https://www.ncbi.nlm.nih.gov/pmc/articles/PMC6631739/ on how to define stillbirths and what to use as denominator, don’t use rate and ratio’s as they are the same.

Response: We avoided using the same terms for different approaches (rate and ratios). Now, every mention was made in accordance with what had been actually estimated (stillbirth ratios when the referenced paper used live births in the denominator and stillbirth rate when they used total births). We also made clear that our analysis considered only stillbirth ratios (the number of fetal deaths per 1,000 live births) and not stillbirth rates (number of fetal deaths per 1,000 total births (live births and fetal deaths).

- Line 78 “3 million maternal, fetal and neonatal deaths….” Is unclear what is 3 million….

Response: We changed the paragraph to make it clearer: “Improvements in periconceptional and pregnancy healthcare can potentially reduce the majority of stillbirths [4-5]; already existing cost-effective interventions can save the lives of more than one million babies annually worldwide [6].”

- Be aware to be consistent in the definition of stillbirth rate (WHO – denominator is the total births)

Response: We have revised and changed all mentions of stillbirth rates and ratios to adequately reflect the indicator accordingly.

- Line 97-101 is too long for one sentence and by referring to the article you can summarize what you want to say – stillbirth rates and the annual reduction are very heterogenous on … and then summarize. because all the figures in the introduction make it too long.

Response: Thank you. We changed it accordingly.

- Relate these differences in stillbirth rate in the Latin American Caribbean region with the objective of your study so that we can better understand the relevance of this study.

Response: The following sentences aims to provide justification and relevance for the study: “Understanding the current burden of stillbirths in the region and the factors associated with its occurrence is ultimately important to reduce heterogeneity and improve related policies in the region.

Valuable information on current challenges is provided when stillbirth ratios are monitored in different countries of the region. It can contribute to direct actions to decrease this burden and improve the quality of perinatal health.”

- Line 104 – is it contributing or **could** contribute? Are the countries doing this now? Do you have a reference than where individual countries do that?

Response: Thank you for pointing this out; we changed it to “it can contribute to…”. We added a reference to support the sentence.

3. Material and methods

- Please use the strobe checklist and add more structure to the methods section and provide all information accordingly such as describe the type of study etc. <https://www.strobe-statement.org/checklists/>

Response: Thank you for the suggestion. We filled out the STROBE checklist and attached it to the submission.

- Line 121-142 can be made better understandable with a flowchart or table focusing on 1. Maternal characteristics ( age, ethnicity….) 2. pregnancy characteristics (…..), 3. delivery characteristics (…..)

Response: We moved the detailed description to a new table in the supplementary material (Table S1); we kept the summarized and crucial information in the main text. We described the independent variables separated by topics as suggested.

- Line 146 – stillbirth ratio – and total live birth is used as denominator…. This should be more specific and comparable – so please use WHO criteria so that the data can be compared!

Response: In order to avoid misinterpretation, we made clear in the methods that we used stillbirth ratio, based on the live births (not total births).

- I would suggest, although you don’t have the causes of death to use the ICD-PM to be able to compare data ( <https://pubmed.ncbi.nlm.nih.gov/35972943/> , <https://pubmed.ncbi.nlm.nih.gov/32777997/> ).

Response: Thank you very much for suggesting such interesting and relevant papers. We added a suggestion in the discussion to address this matter:

“Although the WHO has proposed a standardized approach for investigating and reporting fetal deaths, the SIP does not contain the cause attributed to the fetal death (Ref ). Including the cause of fetal deaths in the SIP could contribute to audit the implementation of such standardized audits and reviews of fetal deaths. Implementing a standard classification system and post-mortem investigation would be of great benefit for assessing causes and informing root-cause of deaths, potentially decreasing the proportion of unspecified and unspecific causes and informing valuable information for preventive strategies [17]. There are different classification systems for fetal death causes such as the Aberdeen, Wigglesworth, Recode, Tulip, CODAC, ICD-PM and a structured system developed by the Stillbirth Collaborative Research Network [19,20, ref1 , ref2 ]. Applying such classification systems promotes a standardized determination of causes of death based on clear criteria and the identification of associated factors [19].”

- Maternal morbidity (MNM, PTLC, SMO, MD): who attributed maternal morbidity to a case? Who defined a case as an MNM, PTLC etc? and how accurate is the database on this, who classified the case as …..What is the reason the authors choose this (MNM, PTLC, SMO, MD)

Response: We chose a standardized definition for maternal morbidity as defined by the World Health Organization. The conditions related to the definition of maternal morbidity were provided in the supplementary material (Table S2). The occurrence of related conditions was identified by health professionals and they were reported to SIP by local staff assistants. The research team attributed the different degrees of maternal morbidity according to the identification of each condition based on the dataset.

- Is it possible to group the maternal condition according to ICD-PM groups M1-M5

Response: We would like to acknowledge that we did not intend to address the causes of fetal deaths for this particular analysis and no standardized approach was employed to review the fetal deaths. Therefore, we decide to not use the ICD-PM classification for this analysis.

4. Results

- Line 172, do not mention the definition again

Response: Thank you for pointing this out. We deleted it accordingly.

5. Discussion

- Mention your main results, after line 209 also report the other findings shortly

Response: A brief summary of the main results was added in lines 231-233.

- Discuss than those findings the authors started with geographical differences, try to explain why some countries might have higher stillbirth rates…. Then discuss the relevant maternal (hypertension, diabetes, infection) pregnancy and delivery outcomes

Response: Lines 230-250 addressed regional differences and why some countries might have higher stillbirth rates, while lines 251-291 addressed the other relevant conditions.

- Line 255-260 – we do not have any results on BMI, in the discussion discuss your own findings….

Response: We deleted the discussion based on the maternal BMI.

- One limitation is that there are no causes attributed to the stillbirth cases

Response: We added this limitation to the discussion.

- Line 313 -314 the conclusion that the SIP is a tool of utmost importance is not in the scope of this study

Response: The respective sentence was deleted.
